# Natural Products and Traditional Chinese Medicine in Hepatocellular Carcinoma: From Pharmacological Mechanisms to Clinical Translation

**DOI:** 10.3390/ph19091350

**Published:** 2026-08-26

**Authors:** Jingyi Shen, Xiaoya Liu, Xuanyan Yan, Tao Zhang, Xianfang Zhang, Huiquan Gu, Weimin Chen, Zhengwen Wang, Qiang Liu

**Affiliations:** 1 Key Laboratory of Tropical Translational Medicine of Ministry of Education, College of Basic Medical Sciences, Hainan Academy of Medical Sciences, Hainan Medical University, Haikou 571199, Chinahy0206171@muhn.edu.cn (W.C.); 2 The First Department of Clinical Medicine, Chongqing Medical University, Chongqing 400016, China; 3 Department of Pharmacology, College of Basic Medical Sciences, Hainan Medical University, Haikou 571199, China; 4 Department of Hepatobiliary and Pancreatic Surgery, Hepatobiliary and Pancreatic Cancer Center, Affiliated Cancer Hospital of Hainan Medical University (Hainan Provincial Cancer Hospital), Hainan Provincial Clinical Medical Research Center for Liver Diseases and Liver Critical Care, Haikou 570311, China; 5 School of Biomedical Sciences, The University of Queensland, Brisbane, QLD 4068, Australia

**Keywords:** hepatocellular carcinoma, natural products, traditional Chinese medicine, pharmacological mechanisms, clinical evidence, pharmaceutical quality, clinical translation

## Abstract

Hepatocellular carcinoma (HCC) remains difficult to control because recurrence, impaired hepatic reserve, and treatment resistance limit durable benefit. Natural products and traditional Chinese medicine (TCM) provide resources that range from drug-lead discovery to adjunctive multicomponent therapy. This review integrates pharmacological and clinical evidence for purified compounds, semisynthetic derivatives, extracts, formulas, and delivery systems. It focuses on metabolic reprogramming and redox homeostasis, stress responses and regulated cell death, tumor cell plasticity and vascular remodeling, and the immune microenvironment and host response. Recent studies have strengthened selected mechanistic claims through chemical probes, functional perturbation, and resistance models. Clinical research has concentrated on recurrence control after surgery or minimally invasive treatment and on combinations with transarterial chemoembolization, targeted agents, and immunotherapy. Randomized trials and prospective cohorts suggest potential benefit in specific settings, although product standardization, external validation, and long-term follow-up remain limited. Major translational barriers include uncertain active constituents, inadequate batch comparability, missing tumor-exposure data, and sparse herb–drug interaction studies. Future development should match target validation, pharmacokinetics, safety assessment, and clinical endpoints to each product class and clarify whether a candidate is best positioned as a drug lead, adjunctive therapy, or supportive intervention.

## 1. Introduction

Hepatocellular carcinoma (HCC) accounts for most primary liver cancers and remains a leading cause of cancer-related death [1,2]. Chronic viral hepatitis, alcohol-related liver disease, and metabolic liver disease are major etiologies that create distinct tumor ecosystems and hepatic risks [3,4]. Current guidelines integrate tumor stage, liver function, performance status, and prior treatment when selecting therapy [3,4,5]. Resection, transplantation, ablation, transarterial therapy, radiotherapy, targeted agents, and immunotherapy now support sequential care [3,4,5,6,7]. Nevertheless, tumor heterogeneity, limited hepatic reserve, and adaptation under treatment pressure continue to drive recurrence and resistance.

Natural products and TCM provide two related resources that require different standards of evaluation. Chemically defined compounds and semisynthetic derivatives are suited to target validation, structure–activity analysis, and lead optimization [8,9]. Extracts, polysaccharides, and multicomponent formulas may affect both tumor cells and host responses, but their assessment depends on material authentication, quantitative composition, batch consistency, and identification of active constituents in vivo. Delivery systems alter release and tissue distribution and should therefore be evaluated as new medicinal products. Supportive and hepatoprotective interventions have different clinical aims, primarily involving hepatic reserve, symptoms, and treatment tolerance.

Recent reviews have summarized natural products, endoplasmic-reticulum stress, and autophagy in HCC [10,11,12]. However, mechanistic experiments, product quality, in vivo exposure, and clinical outcomes have rarely been connected within one pharmacological framework. This review integrates experimental pharmacology, clinical research, pharmaceutical quality, and safety evidence. It examines the biological basis, appropriate clinical context, and key translational requirements for different intervention classes.

## 2. Review Scope and Literature Identification

This article is a structured narrative review. PubMed/MEDLINE was searched without a lower date limit through 10 August 2026. Searches combined (“hepatocellular carcinoma” OR “liver cancer”) with (“natural product” OR “traditional Chinese medicine” OR herbal OR botanical OR formula OR extract OR polysaccharide) and terms related to mechanism, pharmacokinetics, safety, interaction, clinical trial, recurrence, or survival. Reference lists of key articles were traced, and journal websites, HCC guidelines, and relevant pharmaceutical-quality documents were checked. English language full texts and reports with an English abstract sufficient for assessment were considered. We prioritized primary HCC studies that reported a defined intervention and mechanistic, in vivo, pharmacokinetic, safety, or clinical outcomes. Reviews and guidance informed background and evaluation standards. Non-HCC studies were retained only when they addressed pharmaceutical quality, interaction risk, or indispensable mechanistic context. Study selection was iterative and based on direct relevance, evidentiary information, and contribution to the review framework. Because the article was designed as a narrative rather than a systematic review, PRISMA flow reporting, formal risk-of-bias scoring, and quantitative pooling were not applied. Instead, the design limitations of each clinical study were assessed individually.

## 3. Pharmacological Mechanisms and Preclinical Evidence

### 3.1. Candidate Discovery, Lead Optimization, and Target Confirmation

Evidence in HCC natural product research ranges from computational screening to direct target capture. A multi-omics study of 187 medicinal plants integrated transcriptomics, proteomics, and deep learning to prioritize five candidates and link their activities to expression patterns involving AKR1B10, HMGCR, and THBS1 [13]. Such approaches can narrow candidate space, but they cannot identify circulating or tumor-exposed constituents or demonstrate protein binding. Computational findings become developable pharmacology only after chemical identity, functional dependence, and target occupancy are progressively established.

A 2026 study of a matrine derivative illustrates a more persuasive development sequence. Matrine was hybridized with a thiophene scaffold to produce B10. The compound showed IC50 values of 4.13–5.79 μM in HCC cells and 64.2% tumor-growth inhibition at 40 mg/kg in a xenograft model. Surface plasmon resonance, cellular thermal shift assays, and drug-affinity responsive target stability supported an interaction between B10 and FGFR3. A subsequent PROTAC probe, K2, induced FGFR3 degradation and linked this target to PI3K/AKT inhibition [14]. This evidence chain is stronger than assigning a target through docking alone, although pharmacokinetic, selectivity, and toxicology studies are still needed to define a development window.

### 3.2. Metabolic Reprogramming and Redox Homeostasis

Lipid synthesis provides another route for lead optimization. Lipophilic amide derivatives of mangiferin reported in 2026 inhibited fatty acid synthase (FASN) activity and reduced HCC cell proliferation, migration, and invasion [15]. Compared with parent mangiferin, these derivatives showed greater lipophilicity and stronger enzyme inhibition, supporting structure–activity optimization around FASN. Animal efficacy and pharmacokinetic data are not yet available. The findings therefore support the value of the natural scaffold as a drug lead but cannot be extrapolated to the source herb or conventional mangiferin preparations.

AMP-activated protein kinase (AMPK) links energetic stress to cell growth. Sophoricoside activated AMPK and suppressed proliferation, invasion, and xenograft growth [16]. The study connected energy sensing with HCC phenotypes, but the cellular IC50 approached 320 μM, and the animal dose was 160 mg/kg. Unbound plasma and tumor exposure should now be measured to determine whether the AMPK effect occurs at achievable concentrations.

Ruangan Lidan decoction altered carbon metabolism through a different entry point. The formula reduced miR-9-5p, restored pyruvate dehydrogenase kinase 4 (PDK4), and inhibited tumor growth and metastasis [17]. Experimental manipulation of the miR-9-5p/PDK4 axis supported functional necessity and connected pyruvate metabolism with metastatic behavior. Translation still depends on identifying circulating active constituents and establishing their independent contribution to this axis.

Methanolic litchi extract inhibited glucose-6-phosphate dehydrogenase and weakened pentose-phosphate pathway support in HCC cells [18]. Because G6PD controls NADPH production, the study connects glucose diversion with redox homeostasis. FASN, AMPK, PDK4, and G6PD regulate lipid synthesis, energy sensing, pyruvate use, and NADPH supply, respectively. Together, these studies show that HCC metabolic reprogramming can be perturbed through several entry points. Responsible constituents, tumor selectivity, and achievable exposure will determine which approaches can advance.

### 3.3. Stress Responses, Autophagy, and Regulated Cell Death

Subcellular distribution can directly alter natural-compound pharmacology. Chemical modification redirected Rhein from broad intracellular distribution toward mitochondria and enhanced mitochondrial RECQL4-associated inhibition and anti-HCC activity [19]. The study provided clear evidence of mitochondrial localization. It also shows that comparisons between parent compounds and derivatives should consider tissue distribution, metabolites, and normal-liver exposure, rather than nominal potency alone.

Ponicidin provides one of the more complete target evidence chains in this review. Proteome arrays and a biotinylated probe identified Kelch-like ECH-associated protein 1 (KEAP1) as a binding protein. Ponicidin stabilized the KEAP1–PGAM5 complex, promoted PGAM5 ubiquitination, increased mitochondrial reactive oxygen species, and induced apoptosis [20]. Structural modeling and xenograft studies further supported the mechanism. Target identification, complex remodeling, and in vivo phenotype were linked, giving this study greater mechanistic strength than pathway-expression changes alone.

A study of Huaier in sorafenib resistance connected autophagic processing with ferroptosis. In sorafenib-resistant Huh7 cells and xenografts, Huaier plus sorafenib increased NCOA4, promoted FTH1 degradation, expanded the ferrous-iron pool, and increased lipid peroxidation. The combination also increased ACSL4 and reduced SCD1 and GPX4. Ferroptosis inhibitors, an iron chelator, and NCOA4 silencing each attenuated the effect [21]. These interventions support a necessary role for NCOA4-mediated ferritinophagy and move the study beyond static marker observations. Huaier remains a complex product, however, and its active constituents, tumor exposure, and pharmacokinetic interaction with sorafenib remain unresolved.

Other studies addressed growth signaling and apoptosis. The Pulsatilla-derived triterpenoid AB4 reduced Notch-, Hes1-, and Hey1-associated signaling and induced apoptosis in HCC cells and xenografts [22]. Oxyresveratrol inhibited HepG2 cell proliferation and was accompanied by lower ESR1 and EGFR transcription [23]. These findings suggest possible effects on estrogen receptor and growth factor signaling. However, the study did not provide protein-binding, target-dependence, or animal evidence. ESR1 and EGFR therefore remain candidate mechanisms rather than confirmed targets.

Autophagic flux must be distinguished from the accumulation of static markers. Acanthopanax senticosus extract reduced Rubicon, arrested cells in G0/G1, and increased autophagic flux [24]. This design is more informative than a single LC3 measurement, but timing, dose, and cell fate remain essential for interpretation. Autophagy can contribute to stress-induced death or sustain resistant cells; standardized flux assays therefore determine the direction of the conclusion [12,25].

Other extract studies reported diverse stress and death phenotypes. Hypnea musciformis initiated a p53-centered transcriptomic and proteomic response in HepG2 cells [26]. Actinidia chinensis constituents induced apoptosis [27], standardized ginger extract reduced oxidative stress, inflammation, and proliferation [28], and Calotropis gigantea fractions inhibited chemical hepatocarcinogenesis and enhanced doxorubicin activity [29]. These studies address established tumors, chemoprevention, and chemosensitization, respectively, and show that natural products can constrain tumor progression through several stress programs.

### 3.4. Tumor Cell Plasticity, Invasion, and Vascular Remodeling

Tumor cell plasticity connects sphere formation, epithelial–mesenchymal transition, and recurrence. Codonopsis pilosula polysaccharides reduced CDK1, modulated PDK1/β-catenin signaling, and inhibited sphere formation, epithelial–mesenchymal transition, and xenograft growth [30]. Several concordant phenotypes support effects on stem-like state and invasive behavior. Defining polysaccharide structure, batch potency, and CDK1 dependence will be necessary to connect the fraction reproducibly with these phenotypes.

Vascular remodeling can be assessed in both tumor and endothelial compartments. Jiedu Fang reduced Aurora A/STAT3/interleukin-8 signaling and inhibited hypoxia-driven angiogenesis [31]. The marine natural product derivative Xg-13 suppressed angiogenesis in endothelial and HCC models; drug-affinity responsive target stability and molecular dynamics analyses supported an interaction with Axl [32]. Together, the studies support modulation of angiogenic phenotypes and provide a pharmacological rationale for antiangiogenic combinations. Such combinations will require parallel evaluation of normal hepatic vessels, bleeding risk, and pharmacokinetic interactions.

Lead optimization and target-dependence screening have produced additional candidates. A urea-containing resveratrol derivative improved metabolic stability, induced G2/M arrest and apoptosis, and reduced metalloproteinases, migration, and invasion [33]. A C20-oxime pachysandra alkaloid analogue showed submicromolar activity in HepG2 cells and inhibited JAK2/STAT3 signaling [34]. Benzophenones from Anemarrhena asphodeloides showed ALDH3A1-dependent activity [35]. Together with B10 and the mangiferin derivatives, these studies illustrate the value of natural scaffolds as drug leads. The pharmacology of optimized molecules cannot be extrapolated to their source herbs or conventional preparations.

### 3.5. Immune Microenvironment and Host Response

The HCC immune microenvironment contains exhausted T cells, tumor-associated macrophages, myeloid-derived suppressor cells, endothelial cells, and activated stroma. Chronic antigen exposure, hypoxia, and nutrient competition jointly weaken antitumor immunity [36]. The central question is therefore not whether one cytokine or PD-L1 changes, but whether an intervention alters immune cell function in an immune-competent setting and improves response to established immunotherapy.

Jianpi Huayu decoction was examined using a design that partly meets this requirement. The formula enhanced PD-1 blockade in HCC models, with changes involving TREM1/DAP12 signaling, tumor-associated macrophages, and natural killer cells [37]. Comparison of immune-competent and immune-deficient models helped distinguish direct tumor effects from host-mediated effects. This design provides more credible preclinical support for formula–immunotherapy combinations than a xenograft alone.

Studies of Xihuang Pills reported changes in STAT3–PD-L1-associated signaling and the tumor immune microenvironment [38]. The findings support immunomodulation, but the evidence remains weaker than studies that include functional immune comparisons. Considered together, the two studies indicate a development sequence for formula-based immunology: establish immune cell function first, then use constituent analysis, loss-of-function, and rescue experiments to identify necessary pathways rather than reducing a complex formula to one target.

Delivery platforms and natural vesicles can also alter macrophages, natural killer cells, the gut microbiota, and short-chain fatty acids [39,40,41]. These effects are informative, but activity depends jointly on the carrier, cargo, and manufacturing process. Such platforms should not be evaluated as ordinary extracts or formulas; Section 5.3 considers them as new medicinal products.

A 2026 Huaier study extended host-response research to the gut–liver axis. Antibiotic depletion and fecal microbiota transplantation indicated that inhibition of orthotopic HCC by Huaier partly depended on the gut microbiota. 16S sequencing and metabolomics further connected Adlercreutzia and its metabolite equol with improvement of the immune microenvironment. In separate experiments, equol inhibited MAPK signaling and induced G0/G1 arrest through the Cyclin E1–CDK2/Rb axis [42]. Microbiota depletion, transfer, and metabolite validation form a relatively complete functional chain. Clinical relevance will depend on whether microbiota context, equol exposure, and Huaier batch potency can be reproduced in patients.

As an additional metabolic candidate, γ-linolenic acid was prioritized against FABP5 using deep learning, docking, and molecular-dynamics analysis. HCC cells showed reduced proliferation, migration, and invasion and changes in death-related phenotypes [43]. The study did not provide biophysical binding, cellular target-occupancy, or animal evidence. At present, the findings support a FABP5-associated candidate mechanism but not a confirmed direct inhibitor.

**Figure 1:**
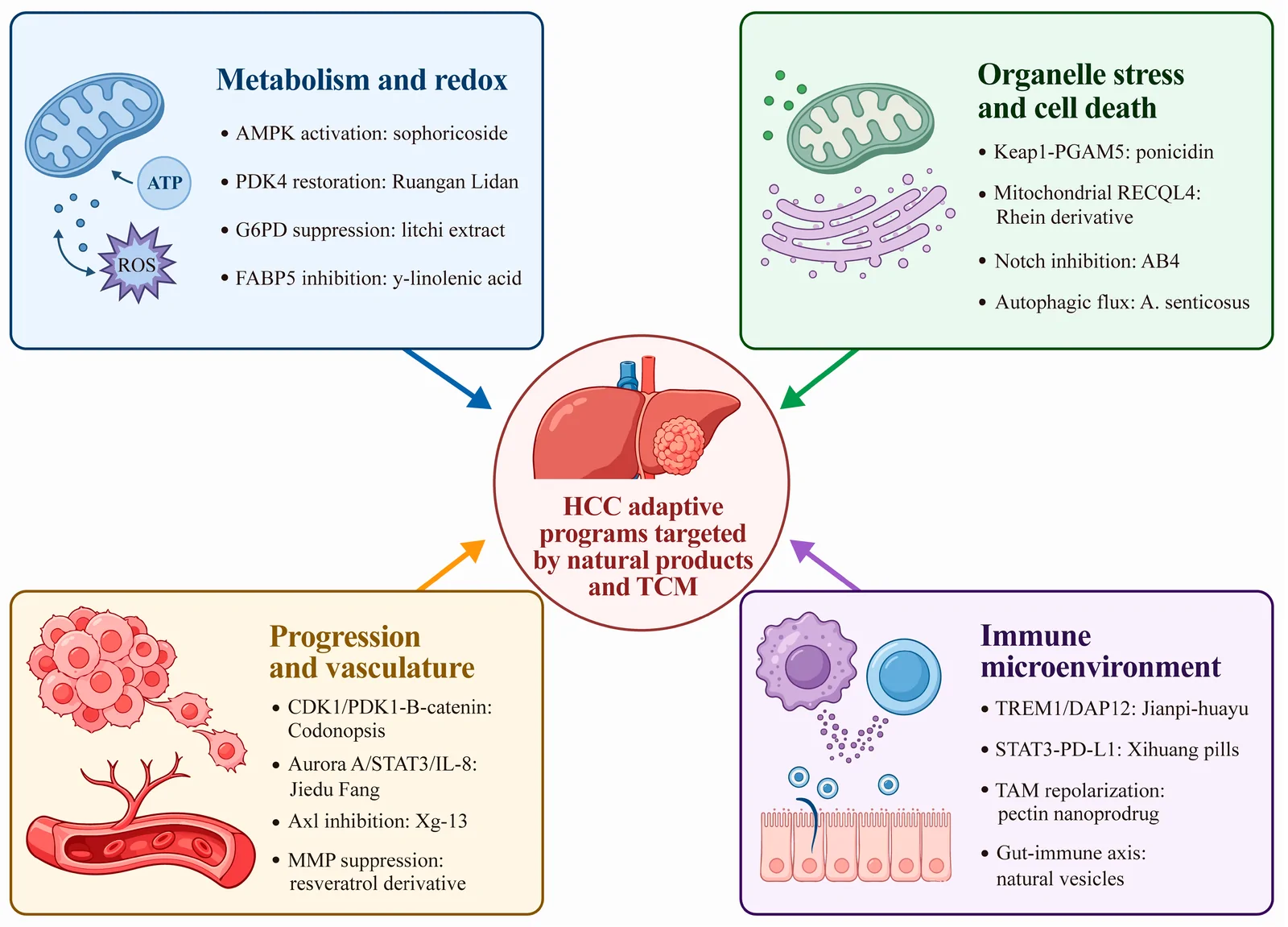
Four adaptive programs engaged by representative natural product and TCM interventions in HCC. Arrows indicate regulatory or functional relationships supported by the source studies; the evidence level for individual relationships is summarized in Table 1.

Figure 1 organizes representative interventions across four programs: metabolism and redox homeostasis, organelle stress and cell death, progression and vascular remodeling, and immunity and host response. The relationships reflect functional entry points proposed and supported to different degrees in the source studies. Table 1 further compares intervention identity, experimental models, mechanistic evidence, major findings, and next validation requirements.

## 4. Clinical Evidence and Therapeutic Roles

### 4.1. Recurrence Control After Curative and Minimally Invasive Treatment

The postoperative Huaier-granule trial provides the clearest current clinical signal [44]. This multicenter, open-label phase IV trial randomized 1044 patients after curative HCC resection at 39 hospitals in a 2:1 ratio. Patients received oral Huaier granules at 20 g three times daily for up to 96 weeks, whereas controls received no further adjuvant therapy. Recurrence-free survival was the primary endpoint, and imaging assessors were blinded.

Mean recurrence-free survival was 75.5 weeks with Huaier and 68.5 weeks in controls, corresponding to a hazard ratio of 0.67 (95% confidence interval, 0.55–0.81). At 96 weeks, recurrence-free survival rates were 62.39% and 49.05%, and overall-survival rates were 95.19% and 91.46%, respectively [44]. The large randomized design, defined product, and prespecified endpoint make the study highly informative. Open treatment, the absence of placebo, and missing plasma exposure–outcome analyses still limit attribution to active constituents and mechanism. The result supports evaluation of this specific Huaier product in the postoperative setting but cannot be extrapolated to all products bearing the same or a related name.

A post-ablation cohort provides contextual support. The study included 340 patients with early HCC, with 170 receiving Huaier after complete thermal ablation and 170 receiving ablation alone [45]. Median progression-free survival was 24.0 and 12.5 months, respectively, with a hazard ratio of 0.67 (95% confidence interval, 0.48–0.94). Overall survival did not differ significantly. The direction was consistent with the postoperative trial, but retrospective allocation and residual confounding make the evidence weaker than randomized data.

A randomized controlled trial of Yangyin Fuzheng Jiedu prescription, published in 2026, provides new evidence for recurrence control [46]. The single-center study assigned 300 patients with BCLC stage 0–A disease in a 1:1 ratio to minimally invasive treatment plus the formula or minimally invasive treatment alone. Local treatment included radiofrequency ablation, TACE, or both. The formula was administered at 150 mL twice daily for 48 weeks. Recurrence-free survival rates at 48 weeks were 84.7% and 74.0%, corresponding to a hazard ratio of 0.54 (95% confidence interval, 0.32–0.90). Adverse event rates were similar, and no grade 3 or higher events occurred. Random sequence generation, sealed-envelope allocation concealment, and blinded imaging assessment strengthened credibility. The investigators also analyzed nine batches of the 11-herb decoction by UHPLC–Q Exactive MS; fingerprint similarities exceeded 0.95 in both positive- and negative-ion modes. Linking clinical outcomes with consistency testing of the investigational product is methodologically valuable. Single-center recruitment, open treatment, heterogeneous local procedures, and short follow-up still require resolution through longer multicenter studies.

### 4.2. Combinations with TACE, Targeted Therapy, and Immunotherapy

Fuzheng Jiedu Xiaoji formulation has been evaluated with transarterial chemoembolization (TACE). A randomized, non-blinded study of 291 patients with HCC reported improved one-year overall and progression-free survival, with stronger effects in selected BCLC subgroups [47]. HPLC–MS/MS, docking, cell experiments, and xenografts were also used to examine AKT/Cyclin D1/p21/p27-associated signaling. The clinical signal merits further study, but independent replication, complete allocation reporting, longer follow-up, and prespecified subgroup analyses are needed.

Clinical and mechanistic findings from the same report answer different questions. Detection of multiple constituents and docking to AKT1 can generate candidate mechanisms, but they do not identify which constituent produced a survival benefit. Benefit after TACE could reflect direct tumor inhibition, altered ischemic response, preserved liver function, or improved treatment completion. Mapping these possibilities separately to radiographic response, survival, hepatic reserve, and treatment-tolerance endpoints is necessary to define the formula’s role in the combination.

A retrospective cohort included 3483 patients with HCC and compared 526 TCM users with 526 nonusers after matching [48]. TCM use was associated with improved five-year survival, with an adjusted hazard ratio of 0.46 (95% confidence interval, 0.40–0.52). The large sample improves signal stability, but treatment selection, immortal-time bias arising from treatment duration, formula heterogeneity, and incompletely measured hepatic reserve may inflate the association. Jianpi Huayu decoction plus PD-1 blockade remains preclinical [37].

In unresectable HCC, a 2025 prospective cohort compared Huaier plus targeted therapy and immune checkpoint inhibition with the same treatment framework without Huaier [49]. The final analysis included 92 patients: 48 in the Huaier group and 44 controls. Median progression-free survival was 8.9 and 5.0 months, respectively, with a hazard ratio of 0.50 (95% confidence interval, 0.32–0.78). Six-month progression-free survival rates were 66.7% and 34.1%. Adverse-event rates did not differ significantly. The signal is directionally consistent with preclinical combination research, but treatment was not randomized, systemic regimens were not uniform, the sample was small, and age differed at baseline. The study can therefore inform a randomized trial but cannot establish the magnitude of benefit added by Huaier to modern systemic treatment.

Natural product combinations with cytotoxic agents also generate dose-optimization hypotheses. Fenugreek-seed extract produced ratio-dependent synergy or additivity with doxorubicin in HepG2 cells [50], and Calotropis fractions enhanced doxorubicin activity in preclinical models [29]. These findings can inform in vivo dose matrices, but in vitro synergy does not demonstrate that dose reduction will be safe. Subsequent experiments should measure antitumor effects together with cardiac, hepatic, and hematologic toxicity.

### 4.3. Supportive Care, Study Protocols, and Evidence Syntheses

Compound glycyrrhizin has primarily been studied for postoperative liver protection. A meta-analysis reported improvements in alanine aminotransferase, aspartate aminotransferase, bilirubin, and albumin [51]. Preserving liver function may improve postoperative recovery, tolerance of subsequent treatment, and treatment continuity and is therefore clinically meaningful. Such benefit should be evaluated using hepatic-reserve and treatment-completion endpoints and reported separately from tumor shrinkage, recurrence, and survival.

The Yangxiao Fukang granule publication is a multicenter, randomized, double-blind, placebo-controlled protocol for stage III hepatitis B-related liver cancer [52]. Planned outcomes include survival, objective response, quality of life, and safety. No efficacy result is available, so the protocol cannot estimate treatment effect. Its value lies in illustrating multicenter recruitment, placebo control, and prospective endpoint definition for a complex formula.

A meta-analysis of Xiao-Chai-Hu decoction combined 54 studies involving hepatitis, liver fibrosis, or HCC [53], but HCC-specific effects could not be extracted independently from the indexed report. An evidence synthesis of Yi Guan Jian decoction included 10 trials and 745 participants and reported composite efficacy, performance status, alpha-fetoprotein, bilirubin, and gastrointestinal outcomes [54]. These syntheses suggest possible directions of clinical benefit while exposing mixed diseases, small samples, inadequate masking, and limited survival data. They are more useful for defining endpoints in the next HCC-specific trial than for replacing product-specific confirmatory studies.

Future trials should follow both HCC endpoint standards and the CONSORT extension for Chinese herbal medicine formulas [55,56]. Randomization, masking, etiology, BCLC stage, Child–Pugh or ALBI status, product composition, dose, adherence, co-medications, and quantitative harms should be prespecified. The 2026 Yangyin Fuzheng Jiedu trial shows that formula studies can use an explicit random sequence, allocation concealment, and blinded imaging assessment. Multicenter replication, longer follow-up, and batch–exposure relationships are the next priorities. Formula effects become interpretable only when study design, product identity, and proposed clinical role are aligned.

Table 2 compares clinical evidence by study design, sample and HCC context, standard treatment, intervention dose, quantitative endpoints, safety, follow-up, and limitations. Randomized trials, cohort studies, meta-analyses, and protocols without results are interpreted separately. Information not reported by the source is marked as NR and was not inferred.

## 5. Pharmaceutical Quality, Exposure, and Delivery

### 5.1. Material Source, Geographic Variability, and Manufacturing Control

Botanical composition is shaped before extraction. Genotype, chemotype, soil, climate, harvest period, plant age, and storage can alter secondary metabolites. A common Chinese name does not define a reproducible medicinal product. WHO guidance on good agricultural and collection practices provides a traceability baseline [57]. Reports should state the accepted Latin binomial, family, medicinal part, origin, harvest information, authentication method, and voucher specimen.

Geographic substitution and processing can further alter chemical profiles. Cultivated and wild material, different geographic sources, and substitutes should be pooled only after chemical and biological comparability has been demonstrated. Solvent polarity, particle size, solid-to-liquid ratio, pH, temperature, time, extraction cycles, and drying method affect yield and composition. Vinegar processing of Euphorbia kansui illustrates that processing may alter both efficacy- and toxicity-related constituents [58].

Chromatographic fingerprints describe overall composition but cannot replace quantitative markers and validated acceptance ranges [59]. Release specifications should cover identity, multi-marker content, contaminants, microbial quality, stability, and batch potency. Good manufacturing practice should also lock critical material attributes and process parameters [60]. A pharmacopeial citation or manufacturer name documents provenance but cannot replace analytical release data for the tested batch.

This requirement directly determines whether clinical evidence can be reproduced. The Yangyin Fuzheng Jiedu trial reported Latin names, medicinal parts, and raw herb doses for 11 ingredients and compared nine investigational batches by UHPLC–Q Exactive MS. Positive-ion analysis identified 22 common peaks and four markers, with similarities of 0.973–0.999. Negative-ion analysis identified 34 common peaks and five markers, with similarities of 0.981–0.995 [46]. This represents progress in reporting batch consistency for a clinical formula, although quantitative constituent ranges, in vivo exposure, and relationships between batch and recurrence remain unestablished. A later study using different materials, processes, or acceptance criteria cannot be assumed equivalent solely because the formula name is unchanged. Table 3 therefore summarizes reported pharmaceutical information for representative complex interventions and explicitly marks missing information as NR.

### 5.2. Pharmacokinetics and Exposure Relevance

Poor solubility, intestinal instability, first-pass metabolism, protein binding, and biliary clearance often restrict effective exposure to natural compounds. Complex formulas add competition during absorption, metabolic conversion, and microbiota-derived metabolites [61]. Pharmacokinetic studies should therefore extend beyond the total plasma concentration and quantify unbound parent compounds and active metabolites in plasma, normal liver, and tumor.

Exposure–effect relationships determine whether a mechanism is pharmacologically achievable. Active cellular concentrations should be compared with unbound tumor exposure in animals or patients and then connected to target engagement or pharmacodynamic markers. When only high-dose xenograft inhibition is available, without constituent exposure or target occupancy, it is impossible to determine whether the formulation, a metabolite, or nonspecific toxicity produced the phenotype.

Borneol altered the exposure and tissue distribution of tanshinone IIA, salvianolic acid B, and ginsenoside Rg1 from a Fufang Danshen preparation in rats [62]. This result cannot be extrapolated to another formula, but it clearly shows that constituents within one product can alter each other’s disposition. Every product proposed for combination therapy requires constituent-level disposition and exposure–effect studies.

### 5.3. Delivery Systems Create New Medicinal Products

Delivery systems can improve poor solubility, rapid metabolism, or low tumor exposure, but they also change release, tissue distribution, and toxicity. The carrier and active constituent together form a new medicinal product. Composition, particle size, drug loading, release, biodistribution, stability, and safety must therefore be characterized independently. Complex nanomedicines rarely reach clinical translation [63]; imaging or tumor-inhibition results cannot substitute for manufacturing reproducibility and comparative toxicology.

A pectin–doxorubicin nanoprodrug used partial oxidation to balance galactose-mediated targeting with doxorubicin loading and was accompanied by macrophage polarization and natural killer cell recruitment [39]. Lipid nanoparticles from Morus nigra leaves combined oral delivery, hepatic uptake, mitochondrial stress, and microbiota modulation [40]. Royal jelly extracellular vesicles affected the microbiota, short-chain fatty acids, immunity, and PI3K/AKT signaling [41]. These immune effects are informative, but cargo analysis, depletion studies, and batch-potency assays are needed to identify the responsible components.

A mitochondria-targeted mesoporous polydopamine system co-delivered evodiamine and IR820 and integrated cRGD tumor homing, triphenylphosphonium-mediated mitochondrial targeting, fluorescence imaging, and photothermal therapy [64]. The strategy for improving evodiamine bioavailability is clear, but multicomponent manufacturing, thermal dosimetry, and component-specific toxicity complicate scale-up. Natural polysaccharides, lipid particles, and extracellular vesicles likewise require structural or cargo comparability, potency, and stability testing [65].

Table 4 compares the design principles and reported advantages of five delivery or subcellular-targeting strategies with the manufacturing, pharmacokinetic, and safety evidence needed for pharmaceutical development. Carrier effects are not treated as equivalent to effects of the corresponding free natural constituent.

## 6. Safety and Herb–Drug Interactions

### 6.1. Pharmacokinetic Interactions

Product-specific interaction data are absent for most reviewed formulas. This is not a distant research issue but a present constraint on combination use. Patients with HCC often receive tyrosine-kinase inhibitors with narrow exposure windows, antiangiogenic agents, immunotherapy, or locoregional treatment. Real-world safety data have documented suspected herb–anticancer drug interactions, although product identity is often incomplete [66,67].

Herbal products can affect CYP enzymes, UGT pathways, P-glycoprotein, and uptake transporters [68,69]. Enzyme inhibition may increase anticancer-drug exposure and dose-related toxicity, whereas induction may lower effective exposure. Transporter changes can also alter tissue distribution. Activation of the pregnane X receptor (PXR) or constitutive androstane receptor (CAR) may cause delayed enzyme induction, so a single in vitro inhibition assay cannot predict clinical direction.

Indirect studies provide mechanistic signals for risk screening. Sea buckthorn altered PXR/CAR signaling and CYP2C function in an inflammatory liver-injury model [70]. Commiphora myrrha resin extract increased CYP2C9 expression through a PXR-associated mechanism in HepG2 cells [71], and selected TCM constituents modulated P-glycoprotein in model systems [72]. These findings do not establish a clinical interaction with a specific HCC regimen. They identify enzymes, receptors, and transporters that warrant priority testing.

Clinical risk assessment must be conducted at the level of the tested product. Studies should integrate reversible and time-dependent inhibition, enzyme induction, active metabolites, protein binding, and transporter effects and compare these findings with expected clinical exposure. Only then can the direction and magnitude of an interaction and its practical effect on efficacy or toxicity be estimated.

### 6.2. Pharmacodynamic and Organ-Specific Risks

Patients with HCC often have cirrhosis, cholestasis, portal hypertension, thrombocytopenia, or impaired synthetic function. Additive liver injury can interrupt effective treatment even without a measurable pharmacokinetic interaction. Herbal and dietary supplements can cause clinically important liver injury, occasionally with severe outcomes [73,74]. Net clinical benefit in a combination regimen must therefore include tumor control, hepatic reserve, and treatment continuity.

Baseline and follow-up monitoring should include bilirubin, aminotransferases, alkaline phosphatase, albumin, international normalized ratio, renal function, and symptoms of decompensation. Suspected product-related liver injury requires withdrawal of the suspect product, exclusion of competing causes, and structured causality assessment. Antiangiogenic agents also carry bleeding and thrombotic risks [75], making coagulation and portal-hypertension status particularly important.

Products with antiplatelet, anticoagulant, endothelial, or vasoactive effects require specific assessment during anti-VEGF treatment or invasive procedures. Baseline gastroesophageal varices, platelet count, coagulation, blood pressure, wound status, and recent procedures should be recorded. Immunomodulatory formulas may affect checkpoint efficacy or immune toxicity; a change in PD-L1 expression alone cannot predict the direction.

TACE and chemotherapy add hepatic, cardiac, hematologic, infectious, and wound-healing risks. Combination studies should prespecify these outcomes rather than reporting only total adverse-event counts.

### 6.3. A Product-Specific Interaction Development Program

Interaction testing should begin with a chemically characterized batch and should be repeated after a material manufacturing change. Human liver microsomes, hepatocytes, and transporter systems should assess reversible inhibition, time-dependent inhibition, enzyme induction, UGT activity, PXR, and CAR. In vivo studies should quantify botanical constituents, active metabolites, and anticancer drugs together and collect pharmacodynamic and safety markers at matched time points.

Early clinical studies require intensive pharmacokinetic sampling, explicit stopping rules for liver injury, coagulation monitoring, and complete co-medication records. A negative result applies only to the tested product, batch, dose, schedule, and patient population. This product-specific pathway can convert interaction risk from a general warning into actionable clinical information.

## 7. From Pharmacological Discovery to Clinical Translation

### 7.1. Mechanistic Validation

Network pharmacology and molecular docking can generate hypotheses for multicomponent formulas but cannot demonstrate direct binding alone. Direct-target claims require orthogonal binding or occupancy assays together with loss-of-function and phenotypic rescue [76,77]. Ponicidin approaches this standard through target capture and complex analysis [20], whereas the B10 study used SPR, CETSA, DARTS, and a PROTAC probe to confirm FGFR3 [14]. For complex formulas, a more realistic sequence is to identify circulating or tumor-exposed constituents before testing whether they are necessary for key phenotypes.

In vivo tumor inhibition increases biological relevance but does not automatically convert pathway association into direct target evidence. Likewise, a clinical survival signal cannot validate a protein proposed by docking. Table 1 therefore records binding, functional, pathway, and phenotypic evidence separately and treats in vivo confirmation as another dimension. This approach preserves the value of positive findings while preventing one evidence type from substituting for another.

### 7.2. Biomarkers and Patient Selection

Current candidate biomarkers fall into three groups. The first reflects tumor metabolism, including FASN, AMPK, PDK4/miR-9-5p, and FABP5 [15,16,17,43]. The second reflects immune or host context, including TREM1/DAP12, macrophage state, STAT3/PD-L1, and equol-production capacity [37,38,42]. The third determines treatment tolerance, including hepatic reserve, renal function, and co-medications. The first two groups can generate biomarker–treatment interaction hypotheses, but no clinical study has prospectively selected patients using these markers and completed independent validation.

Personalized assessment in HCC cannot rely on tumor markers alone. A preparation that inhibits a tumor may provide no net benefit if it worsens cholestasis or increases exposure to a targeted drug. Future studies should prespecify assays and cutoffs and integrate molecular subtype, immune state, Child–Pugh or ALBI grade, renal function, co-medications, and interaction risk [78]. Until treatment–marker interactions and external validation are available, biomarkers should support trial stratification rather than routine prescribing.

### 7.3. Models and Clinically Relevant Endpoints

Most preclinical studies use HepG2 or Huh7 cells and subcutaneous xenografts. These models are useful for initial screening but cannot reproduce cirrhosis, portal hemodynamics, immune tolerance, or spatial heterogeneity. Immune studies should use orthotopic, immune-competent, and acquired checkpoint-resistance models. Metabolic studies should measure intratumoral flux, whereas angiogenesis studies should quantify both tumor perfusion and vascular injury in normal liver.

Patient-derived liver-cancer organoids preserve selected tumor features and support comparative drug testing across products [79]. They still lack complete vascular, immune, and cirrhotic contexts and should therefore complement rather than replace in vivo validation. Clinical studies should prespecify and distinguish radiographic response, survival, hepatic reserve, symptoms, quality of life, and treatment completion so that each conclusion maps to the correct endpoint.

Endpoint separation does not diminish the value of supportive care. Liver enzymes, bilirubin, and albumin can reflect recovery and treatment tolerance. Radiographic response and recurrence reflect tumor control, while survival integrates disease and treatment effects. Reporting these outcomes separately identifies whether an intervention is best positioned as a direct antitumor agent, adjunctive therapy, or hepatic supportive treatment.

### 7.4. A Linked Translational Pathway

**Figure 2:**
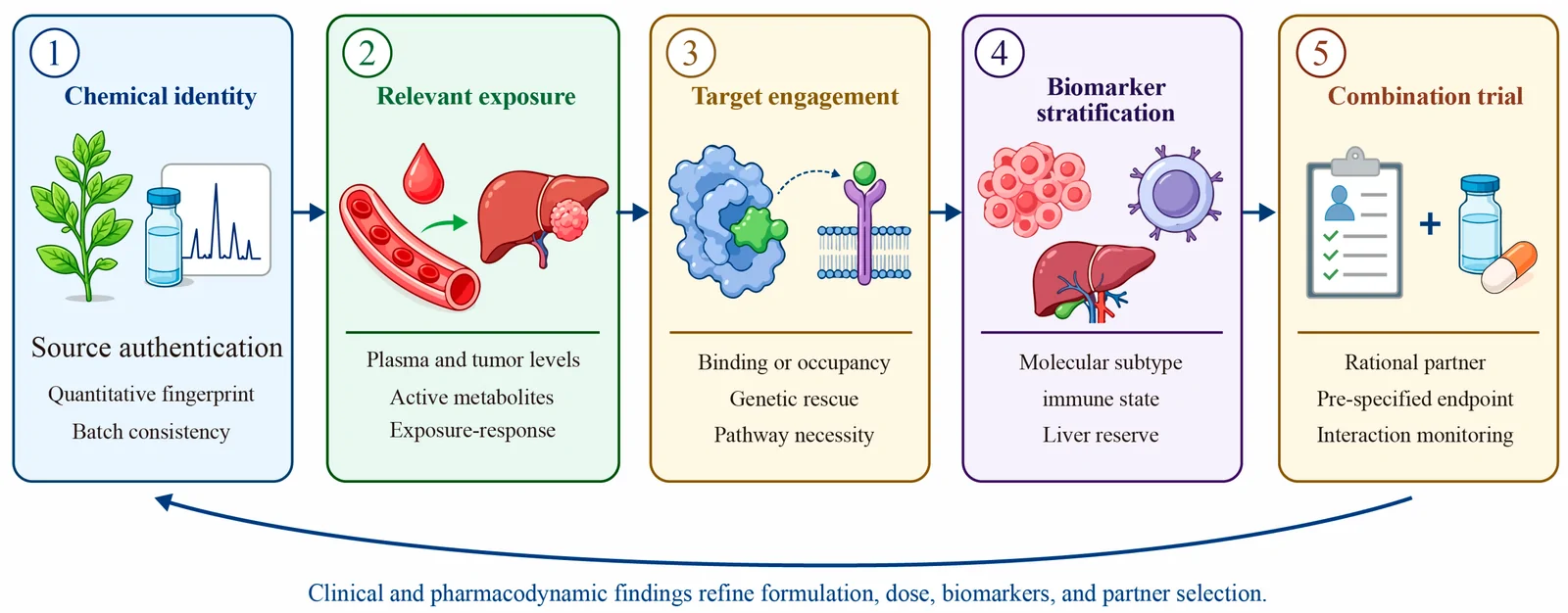
Translational pathway for natural product and TCM research in HCC. Product identity, relevant exposure, target validation, biomarker stratification, and rational combinations form a continuous development sequence. Clinical and pharmacodynamic results should iteratively refine formulation, dose, biomarkers, and combination partners. PK, pharmacokinetics; TCM, traditional Chinese medicine.

Figure 2 summarizes a continuous pathway from pharmacological discovery to clinical validation. Development should begin with an identity-defined, batch-reproducible product. Active constituents or metabolites should then be shown to reach the tumor at relevant concentrations, and a key target or pathway should be demonstrated as necessary for the phenotype. Biomarkers can define potentially responsive groups, whereas combination trials require prespecified clinical endpoints and product-specific interaction monitoring. Clinical and pharmacodynamic results should feed back to formulation, dose, biomarker, and combination-partner refinement. Table 5 converts the main gaps along this pathway into priority studies, minimum qualifying evidence, and decision criteria so that different product classes can be advanced using a common logic.

## 8. Conclusions

As of August 2026, the evidence indicates three promising development paths. First, chemically defined derivatives such as B10 are beginning to confirm targets through orthogonal binding, chemical probes, and functional perturbation, moving natural scaffolds toward testable lead optimization [14]. Second, complex products such as Huaier and Yangyin Fuzheng Jiedu prescription have produced randomized clinical signals after surgery or minimally invasive treatment and have begun to incorporate stronger batch-consistency controls [44,46]. Third, ferritinophagy, microbiota-derived metabolites, the immune microenvironment, and targeted delivery are expanding the pharmacological basis for combination therapy and resistance reversal [21,39,40,41,42,64].

These directions have different levels of maturity. Chemically defined compounds must still show alignment among achievable exposure, target action, and safety margin. Extracts and formulas require stable composition, circulating or tumor-exposed active constituents, and multicenter reproducibility of clinical outcomes. Delivery systems require independent manufacturing, pharmacokinetic, and comparative safety evaluation. Current randomized trials justify further study of recurrence control after surgery or minimally invasive treatment, but they do not support extrapolation to all products sharing a name or to other disease stages.

The value of natural products and TCM in HCC should not be reduced to a generic multi-target claim. Their appropriate roles fall into three categories: drug leads suitable for further optimization, adjunctive interventions combined with standard treatment, and supportive therapies that preserve hepatic function and treatment tolerance. Only studies that define the product, exposure, biological basis, and clinical setting together can convert promising pharmacology into reproducible, comparable strategies with net clinical benefit.

## Figures and Tables

**Table 1:** Models, mechanistic evidence, major findings, and next validation requirements for representative purified compounds and optimized derivatives.

Intervention	Class	Models	Mechanistic Evidence	Main Finding	Next Validation Requirement
Matrine derivative B10 [14]	Matrine–thiophene hybrid	Multiple HCC cell lines; xenograft	Orthogonal binding; chemical probe; in vivo	FGFR3 interaction; PI3K/AKT inhibition; tumor inhibition at 40 mg/kg	PK, kinase selectivity, and systemic toxicology
Lipophilic mangiferin amide derivatives [15]	Optimized xanthone/amide derivatives	FASN enzyme assay; HCC cells	Enzyme inhibition; cellular phenotypes	FASN inhibition; reduced HCC proliferation, migration, and invasion	In vivo efficacy, PK, tumor lipid flux, and safety window
Sophoricoside [16]	Flavonoid glycoside	Huh7/HepG2; xenograft	Functional perturbation; in vivo	AMPK contributes to growth and invasion suppression; tumor inhibition at 160 mg/kg	Link tumor exposure with AMPK effects
Rhein derivative [19]	Anthraquinone derivative	HCC cells; in vivo	Functional dependence; in vivo	Mitochondrial redistribution; RECQL4-associated activity	Compare parent and derivative PK, metabolites, and safety
Ponicidin [20]	Diterpenoid	HepG2; xenograft	Target capture; complex validation; in vivo	Supports KEAP1 interaction; stabilizes KEAP1–PGAM5 complex	Define human-achievable exposure and tumor selectivity
AB4 [22]	Triterpenoid	HepG2/Huh7; xenograft	Pathway association; in vivo	Reduced Notch-associated signaling; apoptosis	Test direct binding or functional necessity of the pathway
Xg-13 [32]	Marine natural product derivative	Endothelial/HCC models	DARTS target stability; molecular dynamics; in vivo	Supports Axl interaction; antiangiogenic activity	Add orthogonal binding or occupancy and assess normal-vessel safety
γ-Linolenic acid [43]	Natural fatty acid	HCC cells	Computational prediction; molecular dynamics; cellular function	FABP5-associated candidate mechanism; direct binding unconfirmed	Test direct binding or occupancy and add in vivo exposure and efficacy
Resveratrol derivative 6k [33]	Optimized polyphenol	HCC cells; in vivo	Phenotypic evidence; in vivo	G2/M arrest, apoptosis, and reduced invasion	Lead PK, selectivity, and comparative safety
Pachysandra alkaloid analogue 7a [34]	Alkaloid analogue	HepG2 cells	Pathway association; cells	JAK2/STAT3-associated apoptosis	In vivo efficacy and off-target profile
Anemarrhena benzophenones [35]	Natural benzophenones	Hep3B/HepG2 cells	Functional target evidence; cells	ALDH3A1-associated anti-HCC activity of anemarrhenone A	The public abstract does not specify the binding method; orthogonal binding, in vivo, and PK validation are needed

Note: ALDH3A1, aldehyde dehydrogenase 3 family member A1; AMPK, AMP-activated protein kinase; FABP5, fatty acid-binding protein 5; FASN, fatty acid synthase; FGFR3, fibroblast growth factor receptor 3; HCC, hepatocellular carcinoma; KEAP1, Kelch-like ECH-associated protein 1; PGAM5, phosphoglycerate mutase family member 5; PK, pharmacokinetics; RECQL4, RecQ-like helicase 4.

**Table 2:** Design, population, treatment, quantitative outcomes, safety, follow-up, and evidence limitations of HCC clinical studies.

Intervention and Design	Population and Clinical Context	Treatment, Dose, and Comparator	Main Endpoints and Quantitative Results	Safety and Follow-Up	Interpretation and Limitations
Huaier granule; multicenter randomized open-label phase IV trial [44]	Randomized n = 1044; analyzed 686 and 316; post-curative resection; BCLC A/B; Child–Pugh A/B; predominantly HBV	Huaier 20 g orally three times daily for up to 96 weeks versus no adjuvant treatment	Mean RFS 75.5 versus 68.5 weeks; HR 0.67 (95% CI, 0.55–0.81); 96-week RFS 62.39% versus 49.05%	96-week OS 95.19% versus 91.46%; periodic SAE monitoring	Large randomized product-specific signal; open treatment, no placebo, and no exposure–outcome link
Huaier granule; retrospective post-ablation cohort [45]	n = 340; 170 per group; early HCC after complete thermal ablation	Huaier after ablation versus ablation alone; dose NR in indexed abstract	Median PFS 24.0 versus 12.5 months; HR 0.67 (95% CI, 0.48–0.94); OS HR 0.76 (95% CI, 0.54–1.07)	Median follow-up 32.5 months; mild gastrointestinal reactions	Direction consistent with randomized trial; retrospective allocation, residual confounding, and nonsignificant OS
Yangyin Fuzheng Jiedu prescription; randomized open-label RCT [46]	Randomized n = 300; all included in ITT; BCLC 0–A; after RFA, TACE, or both; 272 had 48-week outcomes	Formula 150 mL twice daily for 48 weeks versus minimally invasive treatment alone	48-week RFS 84.7% versus 74.0%; HR 0.54 (95% CI, 0.32–0.90); log-rank *p* = 0.016	AE 18.7% versus 14.7%; no grade ≥ 3 AE; 48-week follow-up	Random sequence, allocation concealment, and blinded imaging; single center, open label, heterogeneous local treatment, and short follow-up
Fuzheng Jiedu Xiaoji formulation; randomized non-blinded study [47]	n = 291; HCC receiving TACE; BCLC A–C; predominantly HBV; hepatic reserve NR	Formula plus TACE versus TACE; 48 weeks; formula dose NR in indexed report	Improved one-year OS and PFS reported; complete estimates and CIs unavailable	Incomplete adverse-event reporting; 48 weeks	Clinical and mechanistic results require separation; allocation, masking, liver stratification, and subgroup reporting incomplete
Adjunctive TCM; retrospective cohort [48]	Total n = 3483; matched 526 versus 526; HCC; matched by age, stage, diagnosis period, and treatment type	Heterogeneous adjunctive TCM versus no TCM; variable duration	Adjusted five-year survival HR 0.46 (95% CI, 0.40–0.52); median OS 37.0 versus 9.23 months	Limited quantitative reporting of follow-up and adverse reactions	Large but associative; treatment selection, immortal-time bias, formula heterogeneity, and residual confounding
Huaier plus targeted therapy and immunotherapy; prospective cohort [49]	Final n = 92; unresectable HCC; 48 Huaier and 44 controls; predominantly BCLC C	Huaier 20 g three times daily plus a targeted agent and ICI versus the same treatment framework without Huaier	Median PFS 8.9 versus 5.0 months; HR 0.50 (95% CI, 0.32–0.78); six-month PFS 66.7% versus 34.1%	No significant AE difference; common hypertension, proteinuria, liver dysfunction, and diarrhea	Nonrandomized, small, heterogeneous systemic regimens, baseline age difference, and residual confounding; supports a confirmatory trial only
Compound glycyrrhizin; meta-analysis [51]	18 studies; primary and metastatic liver cancer; total n and liver status NR in abstract	Compound glycyrrhizin plus conventional care versus conventional care; variable dose and duration	TBIL MD −1.61; albumin MD +2.80; composite efficacy RR 1.66	Adverse-event RR 1.13; heterogeneous follow-up	Supports liver-function outcomes; heterogeneous populations, products, endpoints, and study quality
Yangxiao Fukang granule; multicenter double-blind RCT protocol [52]	Planned n = 216; stage III HBV-related primary liver cancer; Child–Pugh C excluded	Conventional treatment plus granule versus placebo; one sachet twice daily for six months	Primary endpoint: one-year survival; secondary endpoints include ORR, PFS, OS, and quality of life; no results	Safety at baseline and months 3, 6, 9, and 12; six months of treatment plus six months of follow-up	Rigorous design but protocol only; conventional treatment not restricted to one regimen
Xiao-Chai-Hu decoction; mixed-disease meta-analysis [53]	54 studies; 5710 patients with hepatitis, fibrosis, or HCC; HCC-specific n NR	Decoction plus variable standard care versus controls	Favorable pooled HCC outcomes reported; HCC-specific estimates absent from the abstract	Variable adverse-event reporting and follow-up	Mixed diseases and treatments; HCC effect cannot be interpreted independently
Yi Guan Jian decoction; systematic review and meta-analysis [54]	10 trials; n = 745; primary liver cancer; incomplete stage and hepatic-reserve reporting	Original or modified formula plus modern therapy versus modern therapy	Efficacy OR 1.84; KPS MD +7.00; AFP SMD −0.36; TBIL MD −1.52	Gastrointestinal adverse reactions OR 0.53; generally short follow-up	Small China-only trials; limited masking and survival data

Note: AE, adverse event; AFP, alpha-fetoprotein; ALBI, albumin–bilirubin; BCLC, Barcelona Clinic Liver Cancer; CI, confidence interval; HBV, hepatitis B virus; HR, hazard ratio; ICI, immune checkpoint inhibitor; KPS, Karnofsky Performance Status; MD, mean difference; NR, not reported; OR, odds ratio; ORR, objective response rate; OS, overall survival; PFS, progression-free survival; RCT, randomized controlled trial; RFA, radiofrequency ablation; RFS, recurrence-free survival; RR, risk ratio; SAE, serious adverse event; SMD, standardized mean difference; TACE, transarterial chemoembolization; TBIL, total bilirubin.

**Table 3:** Identity, preparation, compositional analysis, dose, and batch/GMP evidence for representative complex interventions.

Intervention; Identity/Part	Preparation	Composition and Analytical Control	Experimental or Clinical Dose	Batch/GMP Evidence
Huaier granule; medicinal fungus Trametes robiniophila Murr. [44]	Marketed aqueous-extract granules used in the trial; clinical report did not fully describe source, extraction parameters, or tested batch numbers	The report cited an active proteoglycan mixture containing 41.5% polysaccharides, 12.93% amino acids, and 8.72% water; no quantitative fingerprint or acceptance range for trial batches	20 g orally three times daily for up to 96 weeks	Named manufacturer and marketed product reported; no trial-batch release results or constituent–exposure or batch–outcome relationship
Yangyin Fuzheng Jiedu prescription; 11 botanicals [46]	Centrally prepared by the hospital pharmacy; 300 mL per daily decoction in two doses	Latin binomials, medicinal parts, and raw-herb doses reported; fingerprints of nine batches by UHPLC–Q Exactive MS: positive mode, 22 common peaks/four markers, similarity 0.973–0.999; negative mode, 34 common peaks/five markers, similarity 0.981–0.995	150 mL twice daily for 48 weeks	Nine-batch fingerprint consistency reported; no quantitative marker acceptance range, in vivo exposure, or batch–outcome relationship
Ruangan Lidan decoction; 11 botanicals plus oyster; origins and vouchers NR [17]	Water decocted twice; supernatants pooled and concentrated	Raw-material amounts reported; no quantitative fingerprint or release markers	Cells 2–8 mg/mL; mice approximately 53 g raw-herb equivalent/kg/day	No batch comparison or GMP evidence
Ginger; Zingiber officinale Roscoe rhizome [28]	Standardized extract; detailed extraction NR in abstract	Marker identity and acceptance range NR in abstract	75, 150, or 300 mg/kg/day in DEN/2-AAF rats	Batch comparison NR
Calotropis gigantea stem bark; Thailand; voucher 005191 [29]	95% ethanol maceration; dichloromethane fractionation	Calactin quantified by HPLC; reference standard confirmed by HRMS; constituent classes quantified	2.5 or 5 mg/kg intraperitoneally in DEN rats	Source and process well reported; no multibatch equivalence
Codonopsis pilosula polysaccharide fraction [30]	Polysaccharide solution; extraction and purification NR in abstract	Molecular weight, linkage, branching, and marker ranges not reported	Cell and mouse doses NR in abstract	Batch consistency NR
Jianpi Huayu decoction; derived from Sijunzi decoction [37]	Double decoction; spray-dried powder	Transcriptomic and functional assays; chemical release criteria NR	Dose NR in abstract; mouse HCC and co-culture models	Batch comparison NR
Xihuang Pills; proprietary multicomponent formula [38]	Pill preparation; manufacturing details NR in abstract	UPLC–MS/GC–MS constituent annotation; quantitative ranges NR	Dose NR in abstract; cell and mouse HCC models	Batch comparison NR
Fuzheng Jiedu Xiaoji formulation [47]	Multicomponent extract; full process NR in indexed report	HPLC–MS/MS feature annotation; release criteria NR	Clinical dose NR; combined with TACE	Batch and stability data NR
Yangxiao Fukang granule; 13 named ingredients [52]	Commercial granules; one sachet dissolved in 200 mL water	Manufacturer and pharmacopoeial compliance stated; marker assay NR	One sachet twice daily for six months	Source reported; batch assay NR

Note: 2-AAF, 2-acetylaminofluorene; DEN, diethylnitrosamine; GC–MS, gas chromatography–mass spectrometry; GMP, good manufacturing practice; HPLC–MS/MS, high-performance liquid chromatography–tandem mass spectrometry; HRMS, high-resolution mass spectrometry; NR, not reported; UPLC–MS, ultra-performance liquid chromatography–mass spectrometry.

**Table 4:** Design principles, reported advantages, and pharmaceutical-development requirements for delivery strategies.

Platform	Design Principle	Reported Advantage	Required Development Work
Pectin–doxorubicin nanoprodrug [39]	Partial pectin oxidation; galactose targeting	Tumor delivery; macrophage and NK cell remodeling	Release kinetics, reproducibility, and comparative toxicity
Morus nigra leaf lipid nanoparticles [40]	Oral plant-derived vesicle-like particles	Hepatic uptake; mitochondrial and microbiota effects	Botanical traceability, cargo attribution, and batch potency
Evodiamine/IR820 MPDA system [64]	cRGD homing; mitochondrial targeting	Imaging plus chemo-photothermal therapy	Scale-up, component safety, and thermal dosimetry
Royal-jelly extracellular vesicles [41]	Oral natural vesicles	Hepatic accumulation; immune–gut–metabolic effects	Cargo definition, depletion studies, and stability
Mitochondria-targeted Rhein derivative [19]	Chemical organelle targeting	Improved subcellular target access	Human PK, metabolites, and off-target distribution

Note: cRGD, cyclic arginine–glycine–aspartic acid; MPDA, mesoporous polydopamine; NK, natural killer; PK, pharmacokinetics.

**Table 5:** Key gaps, priority studies, minimum qualifying evidence, and decision criteria for future research.

Key Step	Common Current Weakness	Priority Study	Basis for Advancement
Formula standardization	Name or extraction reported without quantitative consistency	Authenticate materials; define fingerprints, markers, potency, and batch ranges	Trial batches have defined identity and analytical equivalence
Exposure relevance	High cell concentrations or animal doses without tumor PK	Measure unbound constituents and active metabolites in plasma, normal liver, and tumor	Observed exposure covers the concentration producing the pharmacological effect
Target claims	Docking or expression changes interpreted as direct binding	Use orthogonal binding or occupancy plus loss-of-function and rescue	Target engagement is directly supported and necessary for the phenotype
Immune mechanism	PD-L1 or cytokines measured only in xenografts	Use immune-competent orthotopic and resistant models	Immune cell function and treatment response are demonstrably altered
Biomarkers	Markers selected after efficacy analysis without external validation	Prespecify a biomarker–treatment interaction and validate independently	A reproducible treatment–marker interaction is prospectively confirmed
Interaction risk	Combination efficacy assessed without enzyme, transporter, coagulation, or hepatic-risk data	Conduct product-specific PK/PD interaction studies	Direction and magnitude are quantified, and risk is acceptable and monitorable
Clinical endpoints	Liver function, symptoms, response, and survival interpreted together	Prespecify tumor control, hepatic reserve, quality of life, and treatment completion according to clinical role	Each clinical claim is supported by its corresponding prespecified endpoint
Natural nanomedicine	Carrier and complex cargo treated as one active ingredient	Profile cargo and test depletion, potency, release, and comparability	Composition, release, and potency are reproducible across batches

Note: PD, pharmacodynamics; PK, pharmacokinetics.

## Data Availability

No new data were created or analyzed in this study. Data sharing is not applicable to this article.

## Abbreviations

ALBI, albumin–bilirubin; AMPK, AMP-activated protein kinase; CAR, constitutive androstane receptor; CYP, cytochrome P450; HCC, hepatocellular carcinoma; PD-1, programmed cell death protein 1; PD-L1, programmed death-ligand 1; PDK4, pyruvate dehydrogenase kinase 4; PK, pharmacokinetics; PXR, pregnane X receptor; TACE, transarterial chemoembolization; TCM, traditional Chinese medicine; UGT, uridine 5′-diphospho-glucuronosyltransferase; VEGF, vascular endothelial growth factor.
